# PREVALENCE OF BACTERIOBILIA IN PATIENTS UNDERGOING ELECTIVE COLECYSTECTOMY

**DOI:** 10.1590/0102-672020180001e1392

**Published:** 2018-08-16

**Authors:** Rafael Soares de OLIVEIRA1, Paula da SILVA, Carlos Alfredo Salci QUEIROZ, Juverson Alves TERRA-JÚNIOR, Eduardo CREMA

**Affiliations:** 1 Federal University of Triângulo Mineiro, Uberaba, MG, Brazil.

**Keywords:** Laparoscopic cholecystectomy, Antibiotic prophylaxix, Bile, Colecistectomia laparoscópica, Antibioticoprofilaxia, Bile

## Abstract

**Background:**

Cholelithiasis is one of the diseases with greater surgical indication. Currently, laparoscopic cholecystectomy is the gold standard in the treatment of cholelithiasis.

**Aim:**

To analyze the culture of bile from patients with cholelithiasis, mainly in the occurrence of brown and mixed stones.

**Methods:**

Was carried out a prospective study with 246 cases with biliary lithiasis who underwent elective laparoscopic cholecystectomy. Bile culture was performed in all. During anesthetic induction the patients received a single dose of intravenous cefazolin 1 g. At the end of the surgery, the gallbladder was punctured, its contents extracted and immediately placed in a sterile 20 ml propylene flask and promptly sent to bacterioscopy with Maconkey and blood agars. Incubation at 37° C for 24 h was carried out. A protocol was elaborated to include the main factors potentially related to cholelithiasis and the possible presence of associated bacterial infection.

**Results:**

Of the 246 patients, 201 had negative bile culture and 45 positive. Of the 45 patients with bacteriobilia, 34 had growth of a single type of bacterium in bile culture and 11 more than one.

**Conclusions:**

It was observed a relationship between bacteriobilia and age, suggesting that age is a risk factor for bacteriobilia. The use of antibiotic prophylaxis in the elderly is therefore recommended.

## RESUMO

**Racional:**

A colecistolitíase é uma das doenças que têm maior indicação cirúrgica. Atualmente a colecistectomia laparoscópica é o padrão-ouro no seu tratamento.

**Objetivo:**

Analisar a cultura da bile de pacientes portadores de colecistolitíase, principalmente na ocorrência de cálculos com pigmentos marrons e mistos.

**Métodos:**

Foi realizado estudo prospectivo de 246 casos de pacientes portadores de litíase biliar, submetidos à colecistectomia laparoscópica eletiva, sendo realizada cultura da bile. Durante a indução anestésica os pacientes receberam dose única de cefazolina 1 g, intravenosa. No final da operação foi puncionada a vesícula biliar, extraído seu conteúdo e imediata colocação em frasco estéril de propileno de 20 ml e prontamente encaminhado para bacterioscopia com semeadura do material coletado em ágar sangue e de Maconkey com posterior incubação em estufa de cultura a 37° C durante 24 h. Foi elaborado um protocolo capaz de englobar os principais fatores potencialmente relacionados à colecistolitíase e a possível presença de infecção bacteriana associada.

**Resultados:**

Dos 246 participantes, 201 tiveram cultura de bile negativa e 45 positiva. Dos 45 pacientes com bacteriobilia, 34 tiveram crescimento de um único tipo de bactéria e 11 mais de uma.

**Conclusões:**

Foi observada relação entre bacteriobilia e a idade, sugerindo que a idade é fator de risco para bacteriobilia. Recomenda-se assim o emprego de antibioticoprofilaxia nos idosos.

## INTRODUCTION

The cholecystolithiasis is one of the diseases with high surgical indication. The gallbladder stones are present in 10% of the western population. The incidence increases with the age and the risk factors for the appearing are: obesity, diabetes, pregnancy, hemolytic diseases and cirrhosis. The relation woman:man is 4:1 during reproductive age and it equals with aging.

Nowadays, laparoscopic cholecystectomy is the standard treatment. Countless tasks shows that it overcomes in results the one for laparotomy. The hospitalization time is shorter, the patient suffers less pain and the recovery is faster, with earlier work return, the costs are basically the same and there are minor complications^12^.

Bacteriobilia can be defined as the colony of bacteria in bile, not being necessarily followed by clinical or laboratory manifestations. The bacterial flora in the biliary tract is considered as inexistent, in normal conditions.

The protection of biliary ducts against colonization of microorganisms and infections are made by some defense mechanisms, for example: anatomic barriers, physical mechanisms (biliary flux and mucus); chemical factors (bile salts) and immunological defenses. When some of this mechanisms fails, the bacteria colonization and the infection can settle. The route of the bacteria to the biliary duct are not completely clarified. Settled the infection, the clinical situation tends to get worse, showing morbidity and mortality.

In some studies, it was observed a percentage increase of positive cultivations in the bile of carriers of cholelithiasis, mainly in the occurrence of gallstones with brown and mixed pigments. When present, it verifies the predominance of bacteria of intestinal flora in the vesicular fluid and or in the vesicle’s wall, with the prevalence of Gram-negative sepsis from *Escherichia sp, Klebsiela sp* and *Enterobacter sp* genders, which were responsible for 60,4% of the isolated bacteria. It was also observed positive and anaerobic Gram-positive bacteria.

It is estimated, nowadays, that 85% of the patients with cholecystolithiasis keep asymptomatic for at least 10 years. In the last two decades, the knowledge and management evolved in a meaningful way, and a lot of clinical and microbiological parameters have been studied, just like the way how antibiotics and defense systems of biliary tract interfere with the evolution of the infections in bile duct^1^ ^,^ ^4^ ^,^ ^10^ ^,^ ^11^.

With this study, the goal is to analyze the bacterial flora present in the bile of the patients with symptomatic cholecystolithiasis submitted to elective cholecystectomy, as well as its clinical correlations.

## METHODS

A prospect study was realized in 369 cases of patients carriers of gallstones during January 2010 until August 2014, the patients were submitted to elective laparoscopic cholecystectomy in the Clinical Hospital of the Federal University of Triângulo Mineiro, Uberaba, MG, Brazil. In all bile cultivation was done. One hundred twenty three were excluded due to a exclusion factor: non-elective operation, acute cholecystitis, acute pancreatitis, choledocholithiasis, neoplasia, cholangitis and vesicle polyp. For the proposed analysis, material collection was made: a) in the anesthetic induction, and afterwards all patients received a single dose of cefazolin 1 g intravenously; b) at the end of the operation, after removal, the gallbladder was punctured and all the content aspirated and placed immediately on a sterile 20 ml propylene bottle and sent to bacterioscopy on blood and MacConkey agars; c) incubation in culture oven at 37° C for 24 h. Bacterial colonies were identified using biochemical proof standardized in the routine of the lab. With the culture’s result, the patients were divided into two groups: bile positive and bile negative. A protocol was made, able to include the main factors potentially related to cholecystolithiasis and the possible presence of associated bacterial infection. In sequence, for each group, the medical records were analyzed, the main signs and symptoms, the results of the laboratory tests, adopted therapeutic, complications, among other factors. Next, obtained informations were compared between the two groups.

### Statistical analysis

The data were transferred to 2010 Microsoft Excel. The statistical analysis and the graphics were made with the Statview program.

## RESULTS

Two hundred and one out of 246 patients (81,70%) had negative bile and 45 (18,29%) positive. The remaining 45 patients with bacteriobilia, 34 had a single bacteria grow in the bile’s culture and 11 more than one. Among the present bacteria, 25% were *Enteroccocus Sp*; 21.42% *Escherichia coli*; 12.5% *Klebsiella pneumoniae*; and 8,92% *Staphylococcus sp*. negative coagulase. Other bacteria founded were: *Acinetobacter baumanii*; *Enterobacter cloacae*; *Klebsiella oxytoca*; *Proteus penneri*; *Proteus vulgaris*; *Pseudomonas aeruginosa*; *Serratia marcescens*; *Staphylococcus aureus*; *Streptococcus SP*; *Streptococcus beta-hemolytic*, all in percentage less than 5%. All bacteria found were sensitive to at least five different tested antimicrobial agents.

In the routine of the lab, not all bacteria are tested for antibiotics; once identified resistant to cefazolin, it was not tested for this antibiotic. In the cases of positivity for *Enteroccocus Sp* and *Pseudomonas aeruginosa*, that are known for being resistant to cefazolin, this cases had antibiogram with cefazolin.

**FIGURE 1:**
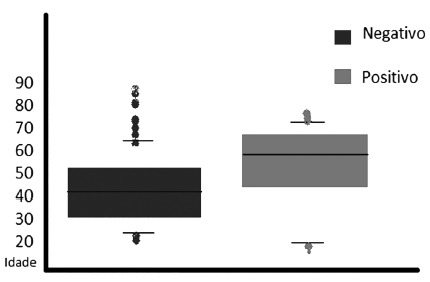
The age of patients and its relation with the positivity (blue for negative and red for positive)

The age was significantly higher in the patients with positive culture (about 56 years) when compared to the negative (about 43 years, p=0.0001, Figure 1). In the positive group, 53% presented age above 50 years, while in the negative the percentage above 50 years was 35.5%

It was observed the relation among the positivity and the following clinical data: systemic arterial hypertension, smoking and diabetes mellitus. The results show that 18 patients were hypertensive and were positive (7.31%). The ones that presented diabetes mellitus and positivity were eight (3.25%). The smokers with bacteriobilia were also eight (3.25%). Among these three comparisons, no significant statistical data were observed (p>0.05) to correlate these clinical data with the bacteriobilia.

In the total sample, 86.58% were women and 13.41% men. This percentage remained when the cultures (negative and positive) were separated. In the negative group 86.56% were women and 13.43% men, and in the positive 86.6% women and 13.3% men).

Other data, such as ultrasound calculi size >0.5 cm (p=0,406) or <0.5 cm (p>0.999) and pathologic findings of the gallbladder (p=0,2583) had no difference in the statistic analysis. 

**FIGURE 2:**
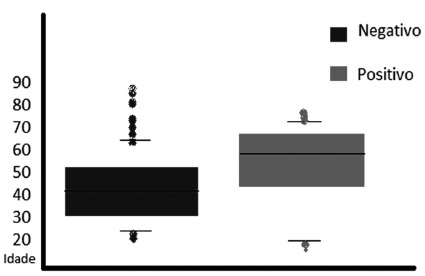
Values of the patients’s leukogram with positive (red) and negative cultures (blue)

The leukogram (Figure 2) had no significant differences when compared the values of the two groups (p=0, 9759)

The hospitalization average time was 1.79 days. Bacteriobilia’s carriers showed 1.84 days of hospitalization and the non-carriers 1.78 days in average.

About the antibiotic prophylaxis used in this study, it was observed that 15 (33.3%) out of the 45 patients with bacteriobilia showed a resistant germ.

## DISCUSSION

The antibiotic prophylaxis is considered a pattern protocol in laparotomic cholecystectomy to reduce the infectious complications; but its use is controversial in laparoscopic cholecystectomy. In a recent meta-analysis, which included 21 randomized clinical trials it was observed that antibiotic prophylaxis reduced the infectious incidence in the surgical site and global infections, just like the time reduction of hospital stay. It is recommended that at least two doses of the antimicrobial - normally cephalosporin - be taken intravenous with the first anesthetic dose.

Recent study showed that the resistance index in patients with age above 65 years is 53.7% to the prophylactic antimicrobial used in laparoscopic cholecystectomy. This could be the influent factor to change the postoperative evolution, especially in high-risk patients.

On the other hand, many prospective studies suggest that the antibiotic prophylaxis is not necessary in elective cases, because the infection rate is low and the prophylaxis do not reduce the incidence of infectious postoperative complications^16^ ^,^ ^17^ ^,^ ^18^. In elective laparoscopic cholecystectomy it not recommended prophylaxis, taking care with the preoperative antisepsis^7^ ^,^ ^15^.

Other studies recommend that only the patients with high-risk should take prophylaxis; for example, advanced age, comorbidities or acute process in the biliary duct. In the present study, it was observed that the age was a relevant factor for the incidence of bacteriobilia and the presence of diabetes mellitus tends to positive bile’s culture. This way, antimicrobial could be used as prophylaxis in elderly people, avoiding its use in low-risk groups; in this way, it would reduce the costs, adverse events and microbial resistance.

Previous studies showed that the judgement based only in clinical observation is not an enough accurate method to guide the presence of bacteriobilia. In the present study, when analyzed the clinical, laboratorial parameters and image exams, significant statistics was not obtained in this matter, not being possible to identify a predictive factor for the infection risk, what matches with another study^8^.

This research had as a significant result, the relation between bacteriobilia and the age, considering that the average age in the positive cultures was 56 years. So, age is suggested as risk factor for bacteriobilia, and in consequence, is recommended the use of antibiotic prophylaxis on elderly people.

The choice of the antibiotic and its dose must be chosen considering the prevalent bacteria, just like the bacterial resistance profile in each institution. It is recommended self-assessment study, in each hospital or region, looking for the best way to use the antibiotic prophylaxis. The application of this study in other services is feasible and can improve the global treatment of the patients, just like reducing the length and cost of hospital stay.

## CONCLUSION

It was observed relationship between bacteriobilia and age, suggesting that age is a risk-factor for it. So, it is recommended antibiotic prophylaxis in elderly people.

## References

[1] Borges MC, Takeuti TD, Terra GA, Ribeiro BM, Rodrigues-Júnior V, Crema E (2016). Comparative analysis of immunological profiles in women
undergoing conventional and single-port laparoscopic
cholecystectomy. Arq Bras Cir Dig.

[2] Edlund YA, Mollstedt BO (1959). Bacteriological investigation of the biliary system and liver in
biliary tract disease correlated to clinical data and microstructure of the
gallbladder and liver. Acta. Chir.Scand.

[3] Gharde P, Swarnkar M, Waghmare LS, Bhagat VM, Gode DS, Wagh DD, Muntode P, Rohariya H, Sharma A (2014). Role of antibiotics on surgical site infection in cases of open
and laparoscopic cholecystectomy a comparative observational
study. J Surg Tech Case Rep.

[4] Kreimer F, Cunha DJ, Ferreira CC, Rodrigues TM, Fulco LG, Godoy ES (2016). Comparative analysis of preoperative ultrasonography reports with
intraoperative surgical findings in cholelithiasis. Arq Bras Cir Dig.

[5] Liang B, Dai M, Zou Z (2016). Safety and efficacy of antibiotic prophylaxis in patients
undergoing elective laparoscopic cholecystectomy: A systematic review and
meta-analysis. J GastroenterolHepatol.

[6] Linhares MM (2001). Fatores de Riso Pré-operatório para Bacteriobilia em Doentes
Portadores de Colecistite aguda calculosa. Rev. Ass. Med. Brasil.

[7] Maya MC, Freitas RG, Pitombo MB, Ronay A (2009). Colecistite aguda: Diagnóstico e tratamento. Revista Abdome Agudo não Traumático.

[8] Matsui Y, Satoi S, Kaibori M, Toyokawa H, Yanagimoto H, Matsui K, Ishizaki M, Kwon AH (2014). Antibiotic prophylaxis in laparoscopic cholecystectomy: a
randomized controlled trial. Plos One.

[9] MoazeniB M, Imani R (2013). Bile bacteria of patients with cholelithiasis and theirs
antibiogram. Acta Med Iran.

[10] Passos MA, Portari-Filho PE (2016). ANTIBIOTIC PROPHYLAXIS IN LAPAROSCOPIC CHOLECISTECTOMY: IS IT
WORTH DOING. Arq Bras Cir Dig.

[11] Rubert CP, Higa RA, Farias FVB (2016). Comparison between open and laparoscopic elective cholecystectomy
in elderly, in a teaching hospital. Rev. Col. Bras. Cir.

[12] Rohde L, Freitas DMOF, Osvald TAB, Bersch VP (2015). Cirurgia videolaparoscópica nas doenças
biliopancreáticas. Rev. Col. Bras. Cir.

[13] Scottish Intercollegiate Guidelines Network (2000). SIGN 45: Antibiotic Prophylaxis inSurgery.

[14] Liang B, Dai M, Zou Z (2016). Safety and efficacy of antibiotic prophylaxis in patients
undergoing elective laparoscopic cholecystectomy: A systematic review and
meta-analysis. J GastroenterolHepatol.

[15] Troyano EB, Castilho JM, Molinos AS, Fernandes LRJ, Oller SB (2015). Bactibilia and Antibiotic Resistance in Elective Cholecysctectomy
An Updated Ecologic Survey. Larchmt.

[16] Yanni FMlP, Morris SG (2013). A selective antibiotic prophylaxis policy for laparoscopic
cholecystectomy is effective in minimising infective
complications. Ann R CollSurg Engl.

[B17] Yan RC, Shen SQ, Chen ZB, Lin FS, Riley J (2011). The role of prophylactic antibiotics in laparoscopic
cholecystectomy in preventing postoperative infection: a
meta-analysis.. JournalLaparoendoscopicAdvSurg.

[18] Turk E, Karagulle E, Serefhanoglu K, Turan H, Moray G (2013). Effect of cefazolin prophylaxis on postoperative infectious
complications in elective laparoscopic cholecystectomy: a prospective
randomized study. Iran Red Crescent Med J.

